# Acute Regression in Young People with Down Syndrome

**DOI:** 10.3390/brainsci7060057

**Published:** 2017-05-27

**Authors:** Clotilde Mircher, Cécile Cieuta-Walti, Isabelle Marey, Anne-Sophie Rebillat, Laura Cretu, Eliane Milenko, Martine Conte, Franck Sturtz, Marie-Odile Rethore, Aimé Ravel

**Affiliations:** Jérôme Lejeune Institute, Paris 75015, France; cecile.cieuta-walti@institutlejeune.org (C.C.-W.); isabelle.marey@institutlejeune.org (I.M.); annesophie.rebillat@institutlejeune.org (A.-S.R.); laura.cretu@institutlejeune.org (L.C.); eliane.milenko@institutlejeune.org (E.M.); martine.conte@institutlejeune.org (M.C.); franck.sturtz@unilim.fr (F.S.); marie-odile.rethore@institutlejeune.org (M.-O.R.); aime.ravel@institutlejeune.org (A.R.)

**Keywords:** down syndrome, adolescence, regression, neurobiology

## Abstract

Adolescents and young adults with Down syndrome (DS) can present a rapid regression with loss of independence and daily skills. Causes of regression are unknown and treatment is most of the time symptomatic. We did a retrospective cohort study of regression cases: patients were born between 1959 and 2000, and were followed from 1984 to now. We found 30 DS patients aged 11 to 30 years old with history of regression. Regression occurred regardless of the cognitive level (severe, moderate, or mild intellectual disability (ID)). Patients presented psychiatric symptoms (catatonia, depression, delusions, stereotypies, etc.), partial or total loss of independence in activities of daily living (dressing, toilet, meals, and continence), language impairment (silence, whispered voice, etc.), and loss of academic skills. All patients experienced severe emotional stress prior to regression, which may be considered the trigger. Partial or total recovery was observed for about 50% of them. In our cohort, girls were more frequently affected than boys (64%). Neurobiological hypotheses are discussed as well as preventative and therapeutic approaches.

## 1. Introduction

Down Syndrome (DS), which is caused by trisomy of chromosome 21, is the first cause of intellectual disability of genetic origin (1/700 births [1]) and is responsible for multiple congenital malformations and medical complications throughout a person’s lifespan. The physician who follows people with trisomy 21 is confronted with two challenges. The first one is to increase the cognitive performances through the use of appropriate supportive measures and the research for a specific treatment. The second challenge is to treat complications that can worsen disability and can occur at any age (West syndrome, sleep apnea, epilepsy, Alzheimer’s disease, etc.). A particularly severe situation is the acute regression that occurs in healthy teenagers.

In recent years, some adolescents and young adults with DS have been described as having rapid cognitive deterioration. This regression is characterized by a loss of autonomy and daily skills, reduced speech, and psychomotor activity. Clinical onset can be sudden or progressive, and the evolution is quite variable. Isolated cases have been reported in the literature since 2011 with various designations [2], including “Down syndrome disintegrative disorder“ [3], “New-Onset Autistic Regression”, “Regression, Dementia, and Insomnia” [3], and “Catatonia” [4].

The etiology of regression remains unknown. In some cases, medical conditions (sleep apnea, Hashimoto’s disease, depression) or stressful life events (end of secondary education, death of a close relative) were noticed prior to regression.

Increased attention from the medical community regarding regression in young adults with DS would improve diagnosis, evaluation, and treatment.

The aim of this article is to share our experience with the report of 30 cases of regression in young DS patients. The Jerome Lejeune Institute (JLI) follows one of the largest cohorts of DS patients (6000 patients aged 0–75 years, with up to 60 years of follow-up). From these retrospective cases, it is possible to describe a syndrome of regression with common symptomatology, age of onset, and evolution.

## 2. Materials and Methods

All patients were followed longitudinally at JLI. Thirty cases of regression were found.

Inclusion criteria:

- 11 to 30 years old.

Psychiatric symptoms with significant regression, from previous state, for language or/and daily skills.

Exclusion criteria:

- Patients with delusion and depression but without regression.
- Patients with severe comorbidities.
- Patients with non-treated objective sleep apnea.
- Patients with profound ID (absence of language or independence).

No patients had a diagnosis of autism before regression.

Patients were born between 1959 and 2000, and were followed from 1983 to the present time.

The clinical evaluation of comorbidities, psychiatric symptoms, autonomy, and language was carried out by the physician of IJL . The diagnosis of associated diseases was confirmed by an expert physician (cardiologist, surgeon, endocrinologist, hematologist, ophthalmologist, etc.) and appropriate complementary tests when necessary.

In the absence of systematic intelligence quotient (IQ) evaluation, the cognitive level was evaluated by the physician according to the level of speech, education, and autonomy as follows: severe ID (partial autonomy, low level of speech, lack of academic skills), moderate ID (autonomy in daily skills, basic language, reading and writing skills), and mild ID (fluent speech and reading, independence outside of the home).

Our results are mainly descriptive. The statistics were calculated from the number of subjects for whom the information was available. (e.g., some explorations are missing for the oldest patients (brain imaging, EEG, anti-thyroid antibodies, and polysomnography). Study data handling was authorized by the French regulation for the protection of personal data and privacy (CNIL—Commission Nationale de L’Informatique et des Libertés).

## 3. Results

### 3.1. Demographic Data and Medical History (Table 1,Table 2,Table 3)

Girls and women represent 64% of this population.

The age of regression seems to be about 20 years; the mean age for regression is 18 years in girls and 21 years in boys. Regarding the number of years of the follow-up, 63% were followed for more than 5 years and near 50% were followed for 10 years or more.

The frequency and repartition of congenital malformation are consistent with previous studies [5]: congenital heart disease (CHD) was the more frequent congenital defect, followed by digestive system anomaly. Thirty percent of the subjects with CHD needed surgery and none had persistent arterial pulmonary hypertension. Thirty percent of the subjects had a thyroid disorder at the time of regression (treated subclinical hypothyroidism for most), but 53% of all patients had a history of thyroid disorder in their life. Regarding surgical history, 27% of the subjects had a tonsillectomy, and 2% underwent cataract surgery. Regarding epilepsy, 13%, i.e., four patients, had epilepsy in their medical history, but none at the time of regression. The first one had a drop attack at the age of 13 years; EEG pattern showed parieto-temporal spikes, and with sodium valproate treatment she became seizure free; regression occurred at the age of 20 years and EEG at this time was normal. The second patient had three tonic-clonic seizures between the age of 7 and 10 years, and was seizure free with sodium valproate; regression occurred at the age of 17 and concomitant EEG was normal at this time. For the third patient, the seizure occurred 10 years after regression; unfortunately, we have no EEG data for him. Finally, the fourth one had tonic-clonic attacks at the age of 45 years, i.e., more than 30 years after regression.

Pre-morbid cognitive level was estimated as severe intellectual disability (ID) (23%), moderate ID (47%), and mild ID (30%).

### 3.2. Clinical Presentation and Evolution

When the information was available (for 57% of the patients), the onset of the regression was acute for 30% and progressive for 70% (but within less than six months). For one patient, there was a history of depression three years before, which was resolved spontaneously.

Trigger event (Table 4): we found a stressful life event that may have triggered regression for a large majority of patients: for 51%, regression coincided with a change of school, whether it be a specialized institution or not. Patients themselves reported being painfully aware of their intellectual disability at the time of the departure or the wedding of a sibling. The illness or death of a relative seemed to be the trigger event for 14%. Overstimulation (i.e., academic and vocational parental ambition) was found in 11% of the patients.

Clinical presentation (Table 5):

Psychiatric disorders: the most frequent symptoms were mood disorders (30%); apathy, extreme slowness or catatonia (37%); and stereotypies (27%). Delusion was reported in four patients, and auto- or hetero-aggressive behavior was reported in 40%. Sleep disorders (initial insomnia, broken sleep) were reported in 10%; loss of appetite was present in two patient and pica in one.

Autonomy: 87% of the subjects presented a decreased independence in daily activities, and 33% presented a total loss of independence. 

Language: speech impairment was observed (94%), as well as mutism (57%). The first sign was often a very feeble voice or a barely audible whisper.

Complementary explorations (Table 6): Brain MRIs were available for 50% of the subjects; 73% of the MRIs were normal. The abnormalities found were brain atrophy or hippocampal abnormalities. Calcification of basal ganglia was found in two subjects. The EEG at the time of regression was normal for 100% of the subjects on which it was performed. Polysomnography was normal in 71%, but performed only on 23% of the patients. Anti-thyroid antibodies (TPO antibody) were analyzed simultaneously on 50% of the subjects and were positive in only three patients (20%). None of the patients had diabetes mellitus or celiac disease. All patients tested (80% of the patients) had negative celiac antibody (endomysium antibody or tissu transglutaminase antibody) either at the time of regression for 13% of the patients, or posteriorly for 57%.

### 3.3. Evolution and Treatment 

Regarding the evolution of the regression (Table 7), 46% did not recover, and some of them even experienced a worsening of symptoms. Forty-three percent partially recovered. The recovery was limited to daily living skills, as 60% of them did not recover their language skills. Several of them were described as very communicative and talkative children, and never talked again for over 10 years after the event, even though they recovered from depressive mood and improved their daily living skills. Finally, 10% were described by their parents as having reached the same state as before the regression.

Most patients received several types of drugs simultaneously or consecutively; benzodiazepines (but not at high doses as recommended for catatonia), selective serotonin reuptake inhibitors (SSRI) (paroxetine, fluoxetine, escitalopram, sertraline), and antipsychotic drugs (haloperidol, propericiazine, amisulpiride for the oldest, and more recently, risperidone and aripiprazole) were the more frequently used. Antiepileptic drugs used as mood stabilizers were less frequently used. One woman needed six electroconvulsive treatment (ECT) sessions and recovered completely. The evaluation of treatment efficacy is impossible in this study, but aripiprazole, risperidone, and paroxetine were reported as the most frequently effective to treat social withdrawal and psychotic symptoms.

## 4. Discussion

The weaknesses of this study are obvious: it is a retrospective study carried out over a long period of time, with different kinds of explorations and no precise data about treatment.

The strengths of this study are the number of patients, the duration of the follow-up with the same outpatients—i.e., over 10 years for nearly 50% of patients—and the clinical data on symptoms and evolution. (The physicians’ experience and the fact that the consultations happened over a long period of time allowed us to carry out a careful clinical examination).

Regarding the demographic data, girls and women represented 64% of the study population, which is quite unusual in the DS population where the male to female sex ratio is 1.23 to 1 [6]. 

Thirty percent of the subjects were evaluated as mild ID before regression, which is probably higher than in the general DS population but needs to be confirmed by prospective studies.

Regarding comorbidity, thyroid disorder is frequent in people with DS (for review: [7]). In the population of DS patients followed at the JLI, 24% from 10 to 20 years of age and 33% from 20 to 30 years of age were treated for hypothyroidism [8], which is similar to this series of patients with regression. The incidence of treated epilepsy is consistent with other studies [9,10].

Children and adolescents with DS have more psychiatric and behavioral disorders (18–38%) than typically developing children [11]. They more frequently present externalizing behaviors such as stubbornness, opposition, inattention, speech problems, poor concentration, and attention-seeking behaviors. Conversely, adolescents and adults with DS show a significant increase in internalizing symptoms (shyness, low confidence, social withdrawal, and depression).

Prasher et al. [12] reported that a significant minority of young adults between the ages of 15 and 30 years with DS present a specific regressive disorder that is “gradual but severe”, characterized by “cognitive regression, language regression, loss of adaptive and social skills and a change in behavior”. 

In recent years, acute regression with cases of catatonia have been reported in adolescents and young adults with DS [4].

This phenomenon in adolescent DS patients was interpreted by some authors as the comorbidity of reactive depressive illness [13,14], mainly before DSM-5 classification which allows the diagnosis of catatonia as an independent disorder. In our study, we presented clinical features of patients as they were described in the medical records at the time of the consultation. Only two out of 30 patients were evaluated after June 2015, when the French translation of DSM 5 was published, and were diagnosed with Catatonia Not Otherwise Specified (NOS). For the patients with slowness or catatonia in Table 5, the diagnosis of catatonia was described as a “catatonic feature” in the clinical report. Probably a significant percentage of the 28 patients who were seen in the last 15 years would now be diagnosed as suffering from Catatonia NOS. The Jerome Lejeune Institute is an exclusively outpatient unit. The treatment of the patients we presented was in most cases previously initiated by their psychiatrist or family physician in their place of residence. 

Individuals with DS or ID do not express emotional distress through the usual channels [14]. Rather than exhibiting sadness or irritability, they often exhibit a more complex presentation with loss of functional skills including speech, continence, and sleep [15]. The diagnosis of depression with atypical presentation is compatible with the psychiatric symptoms and clinical picture found in our patients. However, in the 30 patients reported here, 30% had been evaluated with mild ID, showing fluent reading and speaking skills, before regressing. Furthermore, the evolution in our patients differs significantly from reactive depressive illness, with poor recovery or worsening of symptoms in 46% of cases and full recovery in only 10% of patients, despite the use of different psychotropic drugs. More recently, Akahoshi et al. [16] did a retrospective study of 13 patients with DS with a mean age of 21.2 years at the onset of regression. He found some senile changes in the brain MRI (ischemic changes of the cerebral white matter, hippocampal atrophy, or basal ganglia calcification) even in a patient as young as 10 years old, and a higher incidence of cataract (three patients). Most of the patients of this study partially improved with low doses of psychotropic drugs. He concluded that these acute neuropsychiatric disorders may be related to pre-senile changes, but differed from those presented in typical Alzheimer’s disease with Down syndrome. These earlier Alzheimer’s disease-type pathological changes (e.g., neuronal loss, neurofibrillary tangles, or plaques) were described by Wisniewski et al. [17] in 35-year-old DS patients, followed by the apparition of dementia at a mean age of 54.7 years [18]. These cases of acute regression are different from Alzheimer’s disease because of their earlier onset, their different symptoms and evolution. In our study, brain MRIs were carried out in 50% of patients and were normal in 64% of the cases. In the other patients, we found nonspecific abnormalities; cerebellar hypotrophy and lower hippocampal volume were described in young DS children [19,20] as basal ganglia calcification [21].

Although the prevalence of hypothyroidism at the time of regression is similar to the general DS population, we found that 53% of the patients had a history of hypothyroidism, which is higher than expected. Hashimoto’s encephalopathy is a rare cause of cognitive regression and catatonia [22]. We cannot eliminate a possible immune-mediated mechanism for acute regression. Thyroid antibodies (TPO) were tested for 50% of patients at the time of regression, but were absent in 80% of the cases, which is not compatible with the diagnosis of Hashimoto’s encephalopathy. In a case report of regression in a 19-year-old man with DS [2], several immune analyses were performed (Lyme antibodies, anti-streptolysin O, TPO, antinuclear, and N-methyl-D-aspartate receptor DA antibodies (NMDA), and no abnormalities were found. In a retrospective study, G. Worley et al. [3] described 11 children and adolescents with DS with new-onset autistic regression, dementia, and insomnia. He found a predominance of girls (64%), as we did, and a higher level of TPO antibodies: 10 of 11 cases (91%), compared to only five of 21 (23%) in age-matched control subjects with Down Syndrome (*p* < 0.001). Two of their patients received steroid therapy as prescribed in the case of Hashimoto’s encephalopathy, but showed no improvement. The significance of this increased rate of immune disorders in Worley’s patients as well as the frequency of thyroid disorders in our patient series remains unclear. Nevertheless, the contribution of autoimmune disorders cannot be ruled out as a factor in the regression.

Another cause of regression in patients might be a subclinical epileptic process. In our study, when performed, the EEGs were normal for all patients; however, EEGs should be routinely performed in these patients with regression.

One important limitation of our report is the lack of the results of polysomnography for all patients. In our patients, only 6% had obstructive sleep apnea (OSA), but it was probably underdiagnosed especially among the oldest. Furthermore, we did not include patients with non-treated sleep apnea. Capone et al. [23] reported 25 patients with major depressive episodes with functional decline in 68% of them. Twenty-four (86%) of them had OSA compared to 44% of controls without depression. Results of the treatment for sleep apnea were not reported. In our study, when performed, most of the polysomnography reports were normal, but we cannot exclude the possibility that some of our patients, especially the oldest, had sleep apnea.

Tamasaki et al. [24] reported a 15-year-old DS patient with acute regression who recovered after a treatment of donepezil and selective serotonin reuptake inhibitor (SSRI). He hypothesized that this regression could be a process closely related to the defects of serotonergic and cholinergic systems. The effect of SSRI on acute regression in young adults with DS was also reported (case 2 in the study of Akahoshi et al.) [16]. Neurotransmitters play a critical role in brain development and mood regulation. Das and al. [25] published a review about neurotransmitter abnormalities in DS and specifically in the serotoninergic system which is impaired; Coppus et al. [26] found decreased 5-Hydroxy-Tryptophane (5-HT) levels and increased 5-HT metabolites in DS patients. This serotoninergic system dysfunction may result in an increased vulnerability to depression.

In our study, in most cases we found a triggering factor that can be interpreted as a major stress event for the youth. In the vulnerable brain of DS patients, especially during the period of adolescence, this stress can cause a dramatic decrease of neurotransmitters that may contribute to this acute regression. For our patients, fluoxetine seemed to be more effective than other antidepressant drugs, but prospective studies are needed to answer this question.

A prospective study would also need systematic complementary exploration using EEG, brain imaging, polysomnography, and antibody analysis, as well as psychiatric evaluation (particularly to confirm if there are criteria for catatonia) and cognitive assessment. About 50% of our patients recovered at least partially, which is a positive prospective for them and their families. Nevertheless, several patients never recovered oral speech, even with psychotropic drugs, appropriate psychotherapeutic support, and speech therapy. The reason remains unclear and specific research is the most urgent task.

Finally, the prevention of acute regression would also be based on improving patients’ ability to express their feelings, fears, hopes, and ordinary questions that arise acutely at adolescence, using speech therapy and psychotherapeutic support.

## 5. Conclusions

The objectives of this study were to describe young patients with DS who presented acute regression as adolescents or young adults and its evolution in time; this study is descriptive, and prospective study are warranted, but this could help the elaboration of prospective research and guidelines for the prevention and treatment of this dramatic outcome for DS teenagers.

## Figures and Tables

**Table 1:** Demography.

Number of Subjects	30
Sex	
F	20 (64%)
M	10 (36%)
Cognitive level	
Severe ID	7 (23%)
Moderate ID	13 (43%)
Mild ID	10 (33%)
Age for regression (years)	
12–15	10
15–20	6
20–25	8
25–30	6
Mean for girls	18
Mean for boys	21
Number of years of follow-up	
<2	2
2–5	9
5–10	5
>10	14

**Table 2:** Medical History.

Congenital Abnormality		Medical History		Surgery	
Heart malformation	10 (33%)	Treated hypothyroidism	16 (53%)	Heart	3 (30% of CHD)
Digestive system	1 (3%)	Graves’s disease	2 (6%)	Tonsils	8 (27%)
Clubfoot	1 (3%)	Vitiligo, psoriasis	3 (10%)	Cataract	2 (6%)
Choanal atresia	1 (3%)	Hepatitis B	1	Cholesteatoma	1 (3%)
Hippocampus malformation	1 (3%)	Epilepsy	4 (13%)		
		Treated sleep apnea	2 (6%)		
		Scoliosis	6 (20%)		

**Table 3:** Thyroid status at the time of regression.

Thyroid Status		Thyroid Peroxydase Antibody (TPO)	
Unknown	4	Unknown	15 (50%)
Treated hypothyroidism	8 (31%)	Negative	12 (80%)
Grave’s disease	1	Positive	3 (20%)
Normal	17		

**Table 4:** Trigger event.

Unknown	3
Changing of school, separation from parents	15 (50%)
Awareness of disability, wedding of brother or sister	7 (23%)
Assault	5 (17%)
Illness, death of family member or friend	4 (13%)
Overstimulation	3 (10%)
Viral infection	1
Terrorist attack	1

**Table 5:** Clinical presentation.

Psychiatric Symptoms					
Mood		Behavior		Psychotics symptoms	
Depression, sadness	9 (30%)	Apathy, extreme slowness, catatonia	11 (37%)	Delusion	4 (13%)
Anxiety, phobia, anguish	3 (10%)	Auto-aggressive	6 (20%)	Stereotypies, rocking	8 (27%)
Social withdrawal	6 (20%)	Hetero-aggressive, tantrums	6 (20%)	Baseless laughing	5 (17%)
				Soliloquy	3 (10%)
Autonomy		Language		Other	
No regression	4 (13%)	No regression	2 (7%)	Sleep disorders	3 (10%)
Partial regression (needs help for dressing and meals)	16 (53%)	Partial regression	11 (37%)	Loss of appetite, pica	3 (10%)
Total regression (with urine and fecal incontinence)	10 (33%)	Mutism	17 (57%)		

**Table 6:** Exploration.

Exploration	
Brain Imaging	
No brain imaging	13 (43%)
MRI	15
Normal	11 (64%)
Abnormal	4 (25%)
Thin hippocampus (moderate)	1
Para hippocampal sulcus verticalization (minimal)	1
Cerebellar hypotrophy (moderate)	1
Cortical and cerebellar hypotrophia (moderate)	1
TDM	3
Normal	1
Basal ganglia calcium deposition (minimal and moderate)	2
DAT-Scan	1 (normal)
EEG	
Not Done (ND)	19
Normal	11 (100%)
Polysomnography	
ND	23
Normal	5
Abnormal	2

**Table 7:** Evolution.

Evolution	Number (%)
Worsening	3 (10%)
Stabilization without recovering	11 (37%)
Partial recovering (60% recovered autonomy but never spoke again)	13 (43%)
Total recovery	3 (10%)
